# On the Treatment of Peritonitis

**Published:** 1890-04

**Authors:** 


### KENNEDY (C.) ON THE TREATMENT OF PERITONITIS

Speaking of myself, with thirty years' active practice, I pronounce the opium treatment a miserable failure. All patients do not die under the opium treatment, but 75 per cent of them do.

My plan of treatment is simple, and easily stated. I give at once, and repeat at least every hour, good round doses of saline cathartics, until I get at least eight or twelve good alvine discharges. If the stomach rebels, I give 10 grains of calomel each hour. If I do not hear from the saline or the calomel in four hours, I place the patient on her left side, raise the hips, and employ as an enema 1 oz. of some saline cathartic in half a pint of water, and repeat every hour if necessary. I place compresses, wrung out of well-water, over the abdomen, and if the compresses are thin, I change them frequently. If the pain is intense, I inject under the skin 1-4 grain of morphine, and repeat it if necessary, If after a fair trial of this treatment for 36 hours—after I have procured from 10 to 20 operations from the patient's bowels—the patient is not decidedly better in pulse, in temperature, in tenderness on pressure, and in countenance, open the intestine and let out the gas and fluids, and sew it up. Flush the cavity thoroughly with a solution of corrosive sublimate, and dress the wound antiseptically. Of course there are many details of treatment and operation that you will not expect me to give now—your judgment will suggest them.

Dr. Alonzo Clark, in commenting on a case in which recovery followed a laparotomy for acute peritonitis, says: "If the future supports the practice in this case, acute peritonitis is likely to become a surgical disease rather than a medical." In my judgment, the hour is here. The great progress made in abdominal surgery and antisepsis in the past few years increases the chances of recovery in hopeless cases of acute general peritonitis, certainly fifty per cent.—*Am. Lancet*, Dec., 1889.

